# The Effect of Music on Exercise Intensity among Children with Autism Spectrum Disorder: A Pilot Study

**DOI:** 10.3390/jcm7030038

**Published:** 2018-02-26

**Authors:** Ashley C. Woodman, Emily Breviglia, Yumiko Mori, Rebecca Golden, John Maina, Hannah Wisniewski

**Affiliations:** 1 Department of Psychological and Brain Sciences, University of Massachusetts Amherst, 135 Hicks Way, Amherst, MA 01003, USA; ebreviglia@umass.edu (E.B.); hwisniewski@umass.edu (H.W.); 2 Boston Higashi School, Research Division, 800 North Main Street, Randolph, MA 02368, USA; mori@bostonhigashi.org (Y.M.); john.maina@bostonhigashi.org (J.M.); 3 Boston Children’s Hospital, Laboratories of Cognitive Neuroscience, 1 Autumn Street, Boston, MA 02115, USA; rebecca.golden@childrens.harvard.edu

**Keywords:** autism, autism spectrum disorder, exercise, music, jogging

## Abstract

Children with autism spectrum disorder (ASD) are at risk for obesity, commonly have sleep disorders, and exhibit stereotypic behaviors that disrupt their learning. Vigorous levels of exercise have been shown to ameliorate these issues in children with ASD, but little research exists to provide techniques for motivating children with ASD to engage in exercise. The present study examined the effect of music on exercise intensity in a group of 13 elementary school students with ASD. Data were collected across six days during structured (e.g., verbal and physical prompts) and unstructured (e.g., minimal prompting) exercise periods. During these exercise periods, three music conditions were randomized: no music, slow-tempo music, and fast-tempo music. Exercise intensity, measured in Metabolic Equivalent of Tasks by triaxial accelerometers, was greatest during the structured exercise periods and during the slow music condition. Student characteristics moderated the impact of music condition on exercise intensity, such that students with high levels of adaptive behavior or lower levels of maladaptive behavior displayed greater exercise intensity during the fast music condition.

## 1. Introduction

Occurring in approximately 1 in 68 children in the United States [1], autism spectrum disorder (ASD) is a developmental disorder marked by deficits in social interaction and the presence of restricted, repetitive patterns of behavior, interests, and activities [2]. Challenges in social communication and interaction may include impairments in social–emotional reciprocity, poor use of nonverbal communicative behaviors, and trouble developing, maintaining, and understanding relationships. Restricted, repetitive behavior, interests, and activities may include stereotyped motor movements, insistence on sameness, highly limited interests, and hyper- or hypo-reactivity to sensory input [2]. Many individuals with ASD require significant social and educational support throughout their lives [3].

Along with these core symptoms, people with ASD may also experience problems with balance, gait, postural stability, joint flexibility, and movement speed [4]. Motor impairments are considered “associated” rather than “core” symptoms in individuals with ASD. In a study of 154 children and adolescents with ASD, Ming and colleagues [5] reported the prevalence of hypotonia, motor apraxia, reduced ankle mobility, history of gross motor delay and toe walking. Hypotonia, or low muscle tone, was found to be the most common symptom, present in 51% of the sample. Motor apraxia (34%) and toe walking (19%) were common, while reduced ankle delay and gross motor delay were not (2% and 9%, respectively). With the exception of reduced ankle mobility, the prevalence of the gross motor symptoms was lower among adolescents than children. The mechanism through which ASD causes motor deficits is unclear, however several studies have shown disruption in the transmission of serotonin, dopamine, and gamma-Aminobutyric acid (GABA) [6,7,8,9], which could potentially disrupt motor performance. A standardized movement battery found definite movement impairments in 79% of children with ASD (<5th percentile), with a higher percentage (91%) of motor impairments among children with a comorbid intellectual disability [10]. Although motor difficulties may be present in a large percentage of children and adolescents with ASD, these difficulties may only contribute in part to reduced participation in exercise and sports in this population [4]. 

The core and associated symptoms of ASD make it challenging—but not impossible—for children with ASD to engage in age-appropriate levels of exercise. Pan [11] found that children of this population participate in less physical activity at recess than typically developing children and do not participate in the daily amount of moderate-to-vigorous physical activity recommended by the U.S. Department of Health and Human Services [12]. Similarly, Srinivasan et al. [4] posit that social skill inadequacies, including difficulties with social relationships, sharing interest with others, and reciprocating emotions [13], limit one’s ability to participate in group sports. While the child’s functional limitations may play a role, environmental and family factors seem to be more significant determinants of participation in sports for children with disabilities [14].

### 1.1. Impact of Exercise on Well-Being

Insufficient levels of exercise place children with ASD at risk for a myriad of issues. Previous literature has focused primarily on health-related disorders and suggests that children with ASD are more likely than their typically developing peers to be overweight or obese. Srinivasan et al. [4] reported that 30.4% of their sample of children with ASD were obese, compared to 23.6% of typically developing children of the same age. They also suggested that children with ASD have an increased risk of obesity even compared to children with other intellectual or developmental disabilities. Childhood obesity is a serious concern, given that it is associated with health problems like poor glucose tolerance, raised risk of Type II diabetes, hypertension, social exclusion, depression, and sleep apnea [15]. Furthermore, as childhood obesity progresses to adult obesity, the risk of diabetes, certain cancers, heart disease, and osteoarthritis increases, as well [15]. There are also psychological benefits to exercise, such as reduced stress [16,17].

In addition to the adverse effects described above, deficient levels of exercise may also place children with ASD at heightened risk for sleep disorders and subsequent behavior problems [18,19]. Sleep disorders are highly prevalent in children with ASD [20]. Approximately 50–80% of children with ASD have sleep problems, compared to only 9–50% of children without ASD [21]. Shorter sleep duration has been associated with social skill deficits, stereotypic behavior, and increased autism symptom severity [22]. Stereotypic behaviors can interrupt classrooms, require attention from the educator, and impede learning [4,23]. Thus, finding ways to reduce the frequencies of these behaviors is exigent.

In addition to evidence about the problems associated with insufficient exercise, ample research exists to underscore the positive effects that regular physical activity on obesity, sleep and behavior problems. Lobstein et al. [15] apprise that exercise programs can help prevent childhood and adulthood obesity. Similarly, Brand et al. [18] found that in children with ASD, aerobic exercise increased sleep efficiency, shortened sleep onset latency, and decreased wake time after sleep onset, thereby leading to improvements in mood. Additionally, Lang and colleagues [24] postulate that exercise increases adaptive behaviors and decreases maladaptive behaviors. 

### 1.2. Exercise Intensity

Many studies have found that vigorous-intensity exercise may be more beneficial than moderate-intensity exercise to children with ASD. Mild exercise is defined as between 3.0 and 6.0 Metabolic Equivalent of Tasks (METs) [25] and raises a child’s heart rate to 90 to 120 beats per minute (bpm). Vigorous exercise is defined by METs between 6.1 and 8.8 [25] and may raise a child’s heart rate to 130 bpm or more. For individuals with ASD, vigorous exercise helps to reduce stereotypic behavior, whereas moderate-intensity exercise has little influence [26]. This was found in one study in which children alternated 15 min of ball play and 15 min of jogging each day for 5 days. Levinson and Reid [27] found significant reductions in stereotypic behaviors (motor, vocal/oral, and other) after students with ASD completed 15 min of jogging compared to 15 min of walking. More recently, Srinivasan et al. [4] argued that sustained vigorous intensity exercise leads to reductions in aggressive, self-injurious behaviors and hyperactivity in children with ASD based on a review of the existing literature. It is therefore important to utilize vigorous levels of activity in interventions for children with ASD in order to achieve the benefits of exercise on physical and mental health.

### 1.3. Music and Exercise

Music may promote engagement, interactive communication, and play in individuals withASD [28]. It may also decrease aggressive and disruptive behaviors [29] and increase joint attention and social interaction [30] in children with ASD. Berger [31] found that rhythm based, slow-tempo (60 bpm) interventions helped regulate pacing, reduce repetitive behaviors, and enable focus in children with ASD. However, little research to date has examined the effect of music on exercise intensity among individuals with ASD.

Among typically developing individuals, music has been shown to have an effect on exercise intensity. In a 20-min cycling exercise, motivational and nonmotivational music significantly increased distance traveled, in-task affect, and positive attitudes towards exercising when compared to a 20-min cycling condition during which music was not played [32]. There was no significant difference by type of music. All songs were approximately 140 bpm to reflect 70% of the average age participant’s maximum heart rate. Songs were classified as motivational and nonmotivational based on participant ratings. Similar findings have been reported for exercise on a treadmill. More positive affect was observed during the slow and fast music conditions compared to the condition without any music playing [33]. Both music conditions also significantly increased running speed (adjusted by the participants) and heart rate among participants. 

Similarly, Becker et al. [34] compared the effects of mellow music, frenetic music, and white noise on exercise performance in children. The participants were exposed to a music condition for 1 min before exercising for 2 min. The mellow and frenetic music resulted in significantly higher mileage in children and adults compared to white noise [34]. Taken together, these studies demonstrate the impact of music—low or fast—on affect and exercise intensity.

### 1.4. The Present Study

On average, children with ASD do not engage in sufficient levels of vigorous-intensityexercise [4]. Music has been shown to increase exercise intensity among typically developing children and adults, therefore it is likely that music would have a similar effect on children with ASD, however few, if any, studies have examined this relationship to date. The present study aims to examine the impact of music on exercise intensity among children with ASD during structured and unstructured exercise periods.

## 2. Methods

### 2.1. Participants

The sample included 13 students between the ages of 5 and 13 years (*M* = 9.31, *SD* = 2.25) from a private school for students with ASD, the Boston Higashi School, located in Randolph, MA, USA. To be admitted to this school, a primary diagnosis of ASD is required. Eleven students were male and two students were female. This 5:1 ratio is expected among individuals with ASD [35]. This gender ratio is also in line with the elementary school division in this school more generally (15% female). All of the participants had a comorbid diagnosis of intellectual disability. Students in this school jog daily and are periodically exposed to music while jogging.

### 2.2. Procedures

School administrators mailed a cover letter and consent form home to all parents of students in the elementary school division (*n* = 24). Parents who wished to have their child participate in the study were asked to return signed consent forms to the principal investigator. Teachers were consented and asked to complete a baseline booklet of questionnaires for each participating student. Students were shown a social story about the study and told how they could withdraw their participation verbally or nonverbally. Thirteen of the 24 invited students enrolled in this study, representing a response rate of 54%. This study was approved by the Institutional Review Board at the University of Massachusetts Amherst prior to recruitment and data collection.

The present study spanned six days across a 16-day period in March 2017. On each of the six days of data collection, students participated in their regularly scheduled, structured jogging period for 20 min (9:30–9:50). Students from all classrooms in the elementary school division jogged together in a hallway alongside their teachers. Students ran laps around cones placed at each end of the hallway. A video camera stood behind each cone. Students went to homeroom from 9:50 to 10:25. From 10:25 to 10:45, children enrolled in the study participated in a new, unstructured jogging period for 20 min. During this time, students were asked to jog around a circle of cones in the school gym while teachers encouraged them verbally from a distance. A video camera was placed on both sides of the gym. The purpose of the audio and visual recording was to confirm attendance for each student and to observe if any students left the room or displayed any behaviors that affected their participation. The recordings were also used to count the number of verbal prompts given to the students, to ensure that level of prompting was consistent across music conditions. Students wore accelerometers on their waistband on each of the six data collection days. Teachers put the accelerometers on students after they changed into their uniforms in the morning (approximately 9:15) and removed them after the unstructured jogging period (approximately 10:45). Two practice days were completed prior to data collection to habituate students to the accelerometers and video cameras.

Each of the music conditions (i.e., no music, slow music, and fast music) was implemented on two days of data collection for a total of six days of data collection. The order of the music conditions was randomized, but all conditions appeared once before a condition could be repeated. Music conditions appeared in the following order: slow (day 1), fast (day 2), none (day 3), fast (day 4), none (day 5), slow (day 6). The music condition was held constant on a given day to avoid contamination effects. In other words, if students heard fast music during the structured exercise period from 9:30 to 9:50, they heard fast music again during the unstructured exercise period from 10:25 to 10:45.

Playlists of slow and fast music were created for use during the 20-min exercise periods. Target heart rates for vigorous-intensity exercise were calculated based on guidelines from the Centers for Disease Control and Prevention (CDC). For vigorous intensity physical activity, a person’s heart rate should be 70–85% of their maximum heart rate. Using the average participant age (age 9), the estimated age-related maximum heart rate was calculated as 220 − 9 years = 211 bpm. The 70% and 85% levels were calculated as 211 × 0.7 = 147.70 bpm and 211 × 0.85 = 179.35 bpm, respectively. In other words, music ranging from 147.70 to 179.35 bpm would match the average age participant’s target heart rate for vigorous activity. The fast music playlist was composed of five songs with 144 to 160 bpm to reflect this interval. A slow music playlist was composed of six songs with bpm significantly below this interval (60–80 bpm). Songs on each playlist were shuffled randomly and burned to CDs. Students heard the same playlist in the same order on a given day of data collection (e.g., during the structured and unstructured exercise periods), but the order of the songs was shuffled on the second day of data collection in the same music condition.

During the structured jogging period, teachers used verbal and physical prompts when necessary to keep each student jogging. They started by using verbal prompts that address the group as a whole, and then moved to verbal prompts that address specific students. Verbal prompts included phrases like “keep it up everyone”, “let’s go”, and “come on”. After verbal prompts, they began using physical prompts, starting with the least physical. These prompts included gestures, pointing, touch, physical guidance, and hand-over-hand. This prompting protocol was established by the school long before this study. Teachers were asked to continue using this protocol during the structured jogging period. By contrast, however, teachers were asked to refrain from physical prompts during the unstructured jogging period, unless it was necessary for the safety of a student. Students did not participate in either the structured or unstructured exercise periods if they were not in good physical health (e.g., had a fever, cold, or other significant illness requiring the nurse’s attention), in line with school protocol.

To ensure that the level of verbal prompting was similar across music conditions, a research assistant blind to the study’s hypotheses counted the number of verbal prompts for each video recording. There were two video cameras recording during each exercise period, each from different angles. A second research assistant blind to the study’s hypotheses coded five randomly selected video recordings. The number of prompts coded by the first research assistant was averaged across cameras and compared by music condition.

### 2.3. Measures

#### 2.3.1. Demographics

The students’ gender, age and body mass index (BMI) was collected from the school’s health office with parents’ permission.

#### 2.3.2. Maladaptive Behaviors

Teachers completed the Scales of Independent Behavior Revised (SIB-R) [36]. Teachers indicated if the student exhibited problem behaviors across three areas: internalized (e.g., hurtful to self, unusual or repetitive habits, withdrawn or inattentive behavior), externalized (e.g., hurtful to others, destructive to property, disruptive behavior), and asocial (e.g., socially offensive and uncooperative behavior). Teachers indicated if each of the behaviors occurred in the past six months, as well as the frequency and the severity of the behavior. The frequency was rated from 1 = *never* to 5 = *one or more times per hour.* The severity was measured from 1 = *not serious* to 5 = *extremely serious.* An overall maladaptive behavior score was calculated using standardized algorithms. Bruininks et al. [36] have established reliability and validity. The SIB-R is widely used in studies with children with ASD and has demonstrated good validity and reliability in this population. The total score was used in this study, with higher scores indicating higher levels of maladaptive behavior. Internal consistency in the present sample was α = 0.78.

#### 2.3.3. Adaptive Behavior

Adaptive behavior was measured through teacher reports using the Waisman Activities of Daily Living Scale (W-ADL) [37]. Teachers rated students’ level of independence across 17 activities of daily living (e.g., complete household chores, grooming, prepare simple meals) as 0 = *does not do at all*, 1= *could do but does not or does with help*, or 2 = *independent.* If teachers were unsure about a particular item, they were permitted to consult with residential support staff (if the student was also part of the residential program) or the student’s parents. The W-ADL has established strong internal consistency, criterion, and construct validity for individuals with autism, Fragile-X syndrome, Down syndrome, and intellectual disability of other or unknown origin [37]. The total score was used in this study, with higher scores indicating greater independence in activities of daily living. Internal consistency in the present sample was α = 0.90.

#### 2.3.4. Autism Symptom Severity

Autism symptom severity was assessed by the Autism Spectrum Quotient: Children’s Version (AQ-Child) [38]. The teachers answered 50 questions about each student’s behavior and personality. The questions were created to assess five domains of ASD: social skills, attention switching, attention to detail, communication, and imagination. Teachers rated each question on a four-point scale, from 0 = *definitely agree* to 3 = *definitely disagree* with several items reverse coded*.* The AQ-Child has shown good test–retest reliability and high internal consistency and good construct validity [38]. The total score was used in this study, with higher scores indicating more autistic-like behavior. Internal consistency in the present sample was α = 0.90.

#### 2.3.5. Exercise Intensity

The Omron HJA-750C device (Omron Healthcare, Inc., Kyoto, Japan) was placed on the students’ waistbands. The device is approximately the size and weight of a beeper (1” wide, 1.5” long). It was placed on the rear of the elastic waistband of students’ school uniforms, such that their arms would not touch the device when swung side to side. Students did not appear to be bothered by the devices and the devices did inhibit their natural range of motion. The device attached with two clips. The first is a large clip at the back of the device. The second is a backup clip, which is smaller and attached to the device with a short cord. Each device was calibrated for a particular student, based on their height and weight. The device measures METs based on this calibration at 10 s intervals. METs are often used in research to measure exercise intensity, rather than heart rate, because METs are not affected by psychological factors and emotional arousal, such as anxiety [39]. Two outcome variables were created based on the activity data: (1) average METs during the exercise period and (2) the percentage of 10-s intervals spent in vigorous-intensity activity (6 + METs). Validity data on this particular device is not yet available, however previous models of this accelerometer have been shown to be valid in adults, children and clinical populations.

### 2.4. Analytic Plan

To address the first research question about the impact of music on exercise intensity, a series of one-way repeated measures ANOVAs with Bonferroni post-hoc tests were conducted with music condition (i.e., no music, slow music, and fast music) as the independent variable and exercise intensity as the dependent variable. Parallel analyses were conducted with average METs and the percentage of intervals of vigorous-intensity activity as the dependent variables. Analyses were conducted separately for data collected during the structured and unstructured exercise periods.

To address the second research question about potential moderators of this relationship, the analyses described above were repeated with student characteristics (i.e., age, gender, BMI, adaptive behavior, maladaptive behavior, autism symptom severity) as covariates. In addition to main effects, the interaction of music condition by each of the student characteristics was examined (e.g., music × gender, music × age).

## 3. Results

### 3.1. Preliminary Analyses

One case was missing 7 out of 17 items on the W-ADL. Missing values were replaced with the student’s mean on the complete items. Descriptive statistics on the independent variables are shown in Table 1. Only two students (15%) had BMIs in the obese range (i.e., 25 kg/m^2^ or higher), while the remaining students had BMIs in the normal range. The average BMI for this sample (*M =* 19.58 kg/m^2^) is representative of the average BMI for students in the elementary school division more generally (*M =* 19.49 kg/m^2^).

The amount of time spent jogging varied slightly from day to day. The number of minutes spent jogging ranged from 14.83 to 16.66 min (*M* = 15.71, *SD* = 0.70) during the structured exercise period and from 15.00 to 20.00 min (*M* = 16.03, *SD* = 2.00) during the unstructured exercise period. The number of minutes of jogging did not vary by music condition in the structured exercise period, *p* = 0.25, or unstructured exercise period, *p* = 0.65. It should be noted that the average amount of time spent in vigorous-intensity activity was 16 min per day. In other words, students were not engaging in levels of activity that would be considered harmful or strenuous.

There was very strong internal consistency in the number of verbal prompts coded for a given exercise period, *r* = 0.90, *p* < 0.002. There was strong inter-rater reliability for the two coders, *r* = 0.81. There was no significant difference in the number of verbal prompts by music condition during the structured or unstructured exercise periods.

### 3.2. Effect of Music Condition on Exercise Intensity

Table 2 displays the means for exercise intensity across music conditions and exercise periods. Average METs were significantly higher in the structured period in the no music, slow music (60–80 bpm), and fast music (144–160 bpm) conditions, *p <* 0.001. Similarly, levels of vigorous activity were significantly higher in the structured exercise period across music conditions, *p* < 0.001.

During the structured exercise period, the music condition had a main effect on average METs, *p* = 0.02. Bonferroni post-hoc tests indicated that average METs were significantly higher in the slow music condition compared to the fast music condition, *p* = 0.02 [CI 0.36, 3.46]. During the unstructured exercise period, the effect of music on average METs was significant, *p* = 0.05. Average METs was significantly higher in the slow music condition compared to the no music condition, *p* = 0.04 [CI –0.36, 1.48].

During the structured exercise period, the effect of music condition on vigorous-intensity exercise was approaching significant, *p* = 0.07. During the unstructured period, however, music condition had a significant effect on vigorous-intensity exercise, *p* = 0.04. Vigorous-intensity exercise was significantly higher during the slow music condition compared to the no music condition, *p* = 0.04 [CI –23.18, –0.49].

### 3.3. Student Characteristics

The analyses described above were repeated with student characteristics (i.e., age, gender, BMI, autism symptom severity, maladaptive behavior, adaptive behavior) as covariates. The interaction of music condition and each of the student characteristics was examined for both measures of exercise intensity in both the structured and unstructured exercise periods. With respect to average METs, the effect of music condition differed by levels of maladaptive behavior during the structured exercise period, *p* = 0.04, and unstructured exercise period, *p* = 0.04. There was also a significant interaction of music condition by levels of adaptive behavior, but only during the unstructured exercise period, *p* = 0.01. With respect to vigorous-intensity exercise, the effect of music condition was moderated by levels of adaptive behavior during the unstructured exercise period, *p* = 0.02. The remaining interaction terms were not significant. 

## 4. Discussion

Insufficient exercise may place children with ASD at risk for obesity and related health problems over the life course [4,15]. More immediately, lack of exercise may further exacerbate the sleep difficulties and behavior problems already observed in children with ASD [20]. This study is one of the first, to our knowledge, to use music as a tool to elicit high levels of energy expenditure during exercise in children with ASD. We found that slow music, rather than fast music, motivated children with ASD to jog at greater intensity. The effect of music did differ based on student characteristics, notably levels of adaptive behavior and maladaptive behavior.

Past research has demonstrated that music, particularly fast music, increases exercise intensity among typically developing children and adults. Past research has documented effects on heart rate and running speed [33] as well as distance traveled and in-task affect [32]. Findings from the present study support the motivational role of music in children with ASD—regardless of age, gender, BMI, and autism symptom severity. Contrary to our expectations, students were particularly motivated by slow music. This pattern was consistent across settings (i.e, structured, unstructured) and measures of exercise intensity. Past research has shown that slow music helps children with ASD regulate behaviors and enable focus [31]. Our findings suggest that the positive impacts of slow music on children with ASD extend to exercise intensity as well. Playing slow music in physical education classes, for example, may be a successful strategy to motivate children with ASD to exercise.

**Figure 1:**
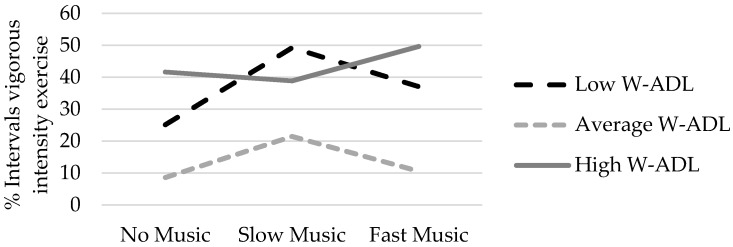
The effect of music condition on vigorous activity as moderated by adaptive behavior during the unstructured exercise period.

To our surprise, the children in this sample were not motivated by fast music, even compared to no music. It is possible that the music selected for the fast tempo playlist was distracting or overstimulating, or that the limited window of observation or sample size was insufficient to detect differences in exercise intensity between fast and no music conditions. An important exception to this observation was for children with fewer limitations in activities of daily living (unstructured period only) and for children with fewer maladaptive behaviors. These children appeared to be motivated by fast music to a greater extent than slow music or no music, in line with expectations for typically developing youth. To illustrate this interaction for vigorous-intensity exercise during the unstructured exercise period, means were plotted for students with low (SD below the mean), average (within SD of the mean) and high (SD above the mean) levels of adaptive behavior (Figure 1). Students with low and average levels of adaptive behavior showed a similar peak in vigorous activity during the slow music condition as compared to the no and fast music conditions. Students with high levels of adaptive behavior, however, showed higher levels of vigorous activity during the fast music condition. The same pattern of results was found for levels of maladaptive behavior. The children with fewer maladaptive behaviors showed a preference for fast music compared to slow music and no music.

A similar pattern was found for the effect of music on joint attention in children with ASD [40]. Among children with severe autism symptoms, simple, predictable music was most effective in evocating joint attention. For children with mild or moderate levels of autism symptoms, complex and variable music was most effective. Future research should further explore the role of individual differences in response to different styles of music.

### Limitations

This study extends the limited literature on the impact of music on exercise intensity among children with ASD, however it is not without its limitations. This sample size is relatively small and lacks gender, racial, and ethnic diversity. There were fewer obese children in this sample (15%) than would be expected based on existing estimates of obesity in children with ASD (30%) and without (24%) [4]. This is likely due to the fact that these children engage in regular exercise as part of their educational curriculum. The extent to which the present findings extend to children with ASD from different backgrounds and exercise levels is unknown. Future research should examine the impact of music on exercise intensity in a broader range of children with ASD.

This study relied on teacher report of maladaptive behaviors, adaptive behaviors and autism symptom severity. Future work should also include parent report on these measures. Another limitation was the inability to make the teachers blind to the music conditions. The tempo of the music may have affected the pace of the teachers running alongside the students. Future studies should attempt to blind the teachers to the music condition (e.g., through noise-canceling headphones) or measure teacher exercise intensity. An additional limitation was the measurement of student BMI approximately 1.5 months prior to the start of data collection. It is possible that students’ BMIs changed in that time frame.

As a final limitation, the songs were selected by the researchers based on their beats per minute. Teachers were given the opportunity to provide feedback on the song choices, but the students were not involved in the song selection. Some research indicates that individuals with ASD may be particularly responsive to music they select, even more so than typically developing individuals [41]. Future research should evaluate the impact of student-selected songs on exercise intensity.

## 5. Conclusions

Findings from the present study have important implications for school-based exercise interventions for children with ASD. Music, particularly slow music, may motivate young students with ASD to engage in the levels of vigorous exercise recommended to avoid obesity and other health problems. Additionally, improved daily activity levels may have immediate effects on sleep difficulties, behavior problems, and academic achievement. Compared to other interventions for people with ASD, jogging is a low-cost, low-risk intervention that can be easily implemented in an educational setting. Music should be incorporated into physical education programs for youth with ASD, just like youth without ASD [42]. Establishing healthy exercise habits in childhood may promote active lifestyles into adulthood [43] and decrease dependence on expensive services.

## Figures and Tables

**Table 1:** Descriptive statistics for independent variables.

Variable	*M*	(*SD*)	Min	Max
Age (years)	9.31	(2.25)	5.00	13.00
Body mass index (kg/m^2^)	19.58	(5.84)	14.60	33.00
Adaptive behavior (W-ADL)	13.83	(5.63)	4.00	22.00
Maladaptive behaviors (SIB-R)	119.08	(10.90)	97.00	133.00
Autism symptom severity (AQ-Child)	86.62	(12.35)	59.00	105.00

**Table 2:** Exercise intensity as a function of music condition and exercise period.

Variable	No Music		Slow Music (60–80 bpm)		Fast Music (144–160 bpm)		Repeated Measures ANOVA
	*M*	(*SD*)	*M*	(*SD*)	*M*	(*SD*)	*F*
Average METs ^b^							
Structured exercise	7.66	(1.91)	8.47 ^a^	(1.35)	6.56 ^a^	(1.92)	5.66 *
Unstructured exercise	4.66 ^a^	(1.25)	5.42 ^a^	(1.51)	4.74	(1.39)	4.14 *
% Time vigorous intensity							
Structured exercise	74.69%	(32.30)	81.73%	(18.34)	59.02%	(34.43)	3.06 ^†^
Unstructured exercise	20.09% ^a^	(18.83)	31.92% ^a^	(24.05)	25.72%	(25.54)	3.82 *

^†^ *p* < 0.10, * *p* < 0.05, ^a^ Bonferroni post-hoc test indicates significant difference in mean. ^b^ Metabolic equivalent of tasks.
